# Systemic Sclerosis: Current State and Survival After Lung Transplantation

**DOI:** 10.7759/cureus.12797

**Published:** 2021-01-20

**Authors:** Artem Minalyan, Lilit Gabrielyan, Shristi Khanal, Bikash Basyal, Chris Derk

**Affiliations:** 1 Internal Medicine, Abington Hospital-Jefferson Health, Abington, USA; 2 Pharmacy, School of Pharmacy, University of Southern California, Los Angeles, USA; 3 Internal Medicine: Rheumatology, Hospital of the University of Pennsylvania, Philadelphia, USA

**Keywords:** systemic sclerosis, scleroderma, lung transplantation, autoimmune

## Abstract

Systemic sclerosis (SSc) is an autoimmune disorder characterized by the involvement of skin and internal organs. With the introduction of angiotensin-converting enzyme inhibitors (ACEIs), scleroderma renal crisis (SRC) is no longer considered a leading cause of death in affected patients. In fact, pulmonary manifestations [interstitial lung disease (ILD) and pulmonary arterial hypertension (PAH)] are currently the major cause of death in patients with SSc. Historically, many centers have been reluctant to offer lung transplantation to patients with SSc due to multiple extrapulmonary manifestations and the assumption of poor post-transplant survival. The purpose of this review is to highlight the recent advances in the evaluation and management of patients with pulmonary manifestations of SSc. We also engage in a systematic literature review to assess all the available data on the survival of patients with SSc after lung transplantation.

## Introduction and background

The word “scleroderma” is derived from the Greek words skleros and derma, which mean “hard” and “skin”, respectively [1]. Systemic sclerosis (SSc) is a chronic autoimmune condition characterized by vasculopathy and fibrotic changes of the skin (scleroderma) and internal organs [2]. Skin manifestations (including their distribution) are the hallmark findings of SSc and are universally used in the classification of SSc: (1) limited cutaneous [lcSSc, also known as CREST syndrome (C - calcinosis, R - Raynaud's phenomenon, E - esophageal dysmotility, S - sclerodactyly, T - telangiectasias)]; (2) diffuse cutaneous (dcSSc: early internal organ involvement, truncal and acral skin manifestations); (3) sine scleroderma (without skin fibrosis but extracutaneous features that most resemble those of patients with lcSSc); and (4) overlap syndrome [many patients with the diagnosis of “mixed connective tissue disease” over time evolve into either SSc or other common systemic autoimmune conditions such as systemic lupus erythematosus (SLE), rheumatoid arthritis (RA), and others] [3].

## Review

Epidemiology

The incidence of SSc has been reported only in several European countries as well as the United States (US) and Canada. It is estimated to range between 0.6 and 2.3 per 100,000 individuals in Europe. Notably, in the US and Canada, the reported incidence is slightly higher, ranging between 1.4 and 5.6 per 100,000 individuals. Similarly, the prevalence of SSc has also been reported only in several European countries as well as the US and Canada. Overall, it ranges from 7.2-9.9 per 100,000 individuals in Norway to 21.3-44.3 per 100,000 individuals in Canada. In the US, the prevalence of SSc has been estimated to be higher in African Americans compared to Caucasians (31.5 vs. 22.5 per 100,000 individuals, respectively) [4].

The diagnosis of SSc is usually made between the third and sixth decades of life. Universally, the female gender has been recognized as a risk factor for the development of SSc. It is worth mentioning that SSc has the highest mortality rate when compared to all other rheumatologic conditions [a standardized mortality ratio (SMR) of 3.39 in a prevalent cohort]. In most cases, the cause of death is related to SSc (predominantly pulmonary manifestations). In addition, patients with SSc have been shown to be at higher risk than the general population of developing different types of malignancy (especially lung cancer and non-Hodgkin lymphoma).

Clinical manifestations

As the name implies, SSc is associated with multiple organ-based manifestations. In patients with diffuse and limited cutaneous subtypes, scleroderma is a hallmark skin manifestation. Despite the variety of cutaneous lesions, their distribution is more significant in the classification of the disease (diffuse vs. limited). Raynaud’s phenomenon (RP) is almost universally present in affected patients and can often have other clinical manifestations. Over time, up to 50% of patients develop irreversible changes in digital arteries leading to ischemic digital ulcers [5]. Interestingly, the presence of anti-Scl-70 antibodies in patients with dcSSc has been associated with a higher risk of developing digital ulcers.

Musculoskeletal manifestations are also very common. Joint pain and contractures of joints due to fibrosis are commonly present in affected patients. Notably, inflammatory arthritis is a rare clinical finding in SSc. When present, it usually has a polyarticular pattern [6].

The majority of patients have gastrointestinal (GI) involvement in SSc. Esophageal dysmotility is the most commonly recognized GI sign. The presenting symptoms may include heartburn, dysphagia, and hoarseness. Gastroparesis is the most common gastric manifestation of SSc. Gastric antral vascular ectasia (GAVE), a cause of chronic upper GI bleeding, which is also known as “watermelon stomach” due to its endoscopic appearance, is also associated with SSc. Intestinal involvement in SSc includes malabsorption, impaired motility, and the development of small intestinal bacterial overgrowth (SIBO) [7].

Renal disease has been widely found in patients with SSc. The course of the disease is mostly benign, except in patients with the development of scleroderma renal crisis (SRC) [8]. The latter usually occurs within the first several years since the onset of the disease. Characterized by the acute onset of renal failure as well as hypertension with features of hypertensive emergency, it is once the most common cause of death in affected patients. Diffuse skin rash, glucocorticoid and cyclosporine use, and the presence of anti-RNA polymerase III antibodies have all been associated with a higher risk of developing SRC.

Cardiac manifestations of SSc can vary and are classified into several categories: (1) those affecting the conduction system; (2) microvascular coronary artery disease; (3) pericardial disease (effusion, pericarditis); and (4) heart failure. The pathogenesis includes the presence of recurrent microvascular spasm leading to ischemia and inflammation as well as cardiac fibrosis [9].

Other systems (pulmonary, nervous, genitourinary) can also be affected in patients with SSc. Of note, an increased risk of certain cancers (lung, hematologic, esophageal, skin), as well as venous thromboembolism, has also been observed in affected individuals [10].

Pulmonary manifestations

Lung involvement is very common in patients with SSc. In fact, it is the second most common organ (after the esophagus) involved in the visceral spread of the disease. With the advent of angiotensin-converting enzyme inhibitors (ACEIs), the leading cause of mortality in patients with SSc shifted from SRC (from 42% to 6%) to pulmonary causes [11]. The most common forms of lung involvement include interstitial lung disease (ILD), pulmonary arterial hypertension (PAH), and their combination. PAH and ILD are known to be the leading causes of mortality in SSc patients, accounting for 28% and 33% of deaths, respectively [12]. Other forms of the disease, although less common, can be categorized into those related to pulmonary vasculature (venous thromboembolism, pulmonary capillary hemangiomatosis, pulmonary veno-occlusive disease), pleural involvement (pleural effusion, pneumothorax), those associated with esophageal dysmotility [recurrent aspiration leading to bronchiolitis obliterans syndrome (BOS) and bronchiectasis], and lung cancer [13].

Interstitial Lung Disease

Patients with dcSSc are known to have a higher risk of developing ILD in the early stages of the disease when compared to patients with lcSSc. The reported prevalence of ILD in SSc varies and is estimated to be close to 50%. The common risk factors include the presence of dcSSc, older age, African American ethnicity, the presence of anti-Scl-70 (anti-topoisomerase) antibodies, and the absence of anticentromere antibodies [14]. In most cases, patients present with cutaneous findings prior to the onset of ILD. In rare instances, ILD-related manifestations (exertional dyspnea, cough, fatigue) can predate other signs and symptoms of SSc. The establishment of the diagnosis of ILD requires prompt clinical suspicion, the performance of pulmonary function tests (PFTs), and diagnostic imaging [high-resolution CT of the chest (HRCT)]. It is worth mentioning that the risk of developing ILD tends to be highest in the early stages of SSc. Hence, it is important to frequently perform PFTs (every four to six months) in the first three years after the diagnosis of SSc is established. It has been reported that low forced vital capacity (FVC) and the progression of fibrosis are independent predictors of mortality [15].

There are no established universal criteria regarding the appropriate time to initiate medical therapy in patients with SSc-ILD. The decision to start therapy depends on the presence of the features associated with the progression of ILD as well as the toxicity profiles of the medications. An increased likelihood of disease progression can be due to one of the following features: (1) the duration of the disease of less than four years; (2) patients with dcSSc subtype; (3) poor pulmonary functional performance (FVC of <65%, diffusing capacity for carbon monoxide (DLCO) of <55%); (4) the progression of ILD on imaging (>20% on HRCT); and (5) the presence of anti-Scl-70 antibodies.

Two commonly used medications for the treatment of SSc-ILD are mycophenolate mofetil (MMF) and cyclophosphamide (CYC) [16]. The Scleroderma Lung Study I (SLS-I) and Scleroderma Lung Study II (SLS-II) were two landmark trials that addressed the use of immunosuppressive medications in patients with SSc-ILD. In the SLS-I trial, the use of CYC for 12 months was associated with improvement of symptoms, quality of life, and FVC percent-predicted when compared to placebo. However, 12 months after cessation of the study, the FVC percent-predicted was found to return to baseline levels in both arms. In addition, more significant side effects (leukopenia and neutropenia) were observed in patients who received CYC compared to the placebo arm [17,18]. In the SLS-II trial, the safety and efficacy of MMF (24 months of therapy) compared to oral CYC (initial 12 months) followed by steroids (final 12 months) were evaluated. No difference in the 24-month FVC percent-predicted was observed between the two groups. In fact, both groups were found to develop clinically significant improvements in their FVC percent-predicted and symptoms. However, leukopenia and thrombocytopenia were more common in patients in the CYC (with steroids) arm compared to the MMF group. Given the above-mentioned findings, MMF is generally preferred as a first-line agent over CYC based on its safety and tolerability profiles. Both CYC and MMF exert anti-inflammatory effects in patients with SSc-ILD. It has been suggested that an additional inhibition of the fibrotic pathway may provide therapeutic benefit in affected patients. Azathioprine (AZA) is not currently used as an initial agent in patients with SSc-ILD. In fact, AZA has never been directly compared to MMF. However, it was found to be less effective (a decline in FVC and DLCO) than CYC in a randomized trial involving 60 patients with early SSc-ILD [19]. As a form of maintenance therapy, MMF is preferred over other agents. It is worth mentioning that the optimal duration of immunosuppressive therapy remains unknown. Biologic disease-modifying antirheumatic drugs (tocilizumab, rituximab) have also been recently used with variable success in patients with SSc-ILD [20,21]. Nintedanib, a tyrosine kinase inhibitor, has been shown to have anti-inflammatory and antifibrotic effects. In the Safety and Efficacy of Nintedanib in Systemic Sclerosis (SENSCIS) trial, the annual rate of decline in FVC was found to be lower in the nintedanib arm when compared to the placebo group [22]. Significantly, soon after the SENSCIS trial results were published, the Food and Drug Administration (FDA) approved nintedanib for use in SSc-ILD. Pirfenidone (PF) is another antifibrotic medication with an unknown mechanism of action. In the LOTUSS trial, the safety and tolerability of PF were demonstrated [23]. In the SLS-III trial (a phase II, multicenter, double-blind randomized study; currently recruiting subjects), the combination of MMF and PF is being compared to MMF with placebo to assess the FVC percent-predicted at baseline and then every three months until the completion of the study (18 months).

Pulmonary Arterial Hypertension

Pulmonary hypertension (PH) is a frequent and heterogenous complication in patients with SSc. PAH is a common cause of PH in affected individuals, accounting for approximately 10% of cases. In comparison to patients with idiopathic PAH, SSc-PAH has a less favorable prognosis and is less responsive to therapeutic interventions. 

Given that SSc affects multiple visceral organs, in addition to PAH [affecting small pulmonary arteries, Group 1 of the World Health Organization (WHO) classification of PH], several other contributing factors to the development of PH have been recognized: (1) the concurrent presence of ILD, which distorts lung architecture (Group 3); (2) cardiac involvement with the development of left ventricular dysfunction and/or myocardial fibrosis (Group 2); (3) pulmonary veno-occlusive disease (PVOD), which has been reported in 61.5% of patients with precapillary PH related to SSc (Group 1); and (4) primary biliary cirrhosis (PBC), the most common liver disorder in patients with SSc, leading to the development of portopulmonary hypertension, a subset of PAH (Group 1) [24]. Some of the common risk factors of the development of PAH include African American ethnicity, older age, telangiectasias, abnormal nailfold capillaries, long-standing disease, and the late onset of the disease.

The diagnosis of PAH can be challenging and requires a high index of suspicion from clinicians. The symptoms are often nonspecific, including dyspnea, exercise intolerance, and generalized fatigue. As mentioned above, PH in patients with SSc may be multifactorial. Therefore, it is important to determine the leading cause of PH. Interestingly, PVOD can have a more acute clinical presentation, and its recognition should prompt early referral for lung transplant (LT) evaluation given poor response to medical therapy [25]. Transthoracic echocardiography is widely used as an initial diagnostic test in patients with suspected SSc-PAH. The measurement of several echocardiographic parameters provides an indirect assessment of the probability of PH: tricuspid regurgitant jet velocity (TRJV) of ≥2.8 m/s, which is used to calculate the estimated pulmonary artery systolic pressure (ePASP), right ventricle/left ventricle basal diameter ratio (>1.0), flattening of the interventricular septum, early diastolic pulmonary regurgitant velocity of >2.2 m/s, and others. Right heart catheterization (RHC) is the gold standard test in the diagnosis of PH. It is usually required to confirm, quantify the severity, and identify the etiology of PH. Interestingly, in 2019, the 6th World Symposium on Pulmonary Hypertension (WSPH) Task Force proposed to use a new diagnostic cutoff of a mean pulmonary arterial pressure (mPAP, supine at rest) of >20 mmHg (previously ≥25 mmHg) to define PH [26].

In addition to the diagnostic challenges of PH, its management also requires the determination of the leading cause of PH to employ a more targeted therapeutic approach. In all patients with PH, functional class and risk stratification should be identified to further decide whether therapeutic interventions (including both monotherapy and combination therapy) should be pursued. The WHO functional classification of PH is similar to other functional classes [for instance, the New York Heart Association (NYHA) classification of heart failure]. Thus, patients with any functional limitation of PH belong to class I. In contrast, patients with class IV PH have symptoms even at rest. In patients with class I PH, both observation and monotherapy [endothelin receptor agonists (ERAs), phosphodiesterase 5 inhibitors (PDE5Is)] approaches are commonly used. Given that the SSc-PAH is a progressive disease, the early initiation of monotherapy may be preferred [27]. In symptomatic patients with the WHO functional class II and III (slight vs. marked limitation of physical activity), a combination therapy is often recommended: an ERA and a medication affecting nitric oxide (NO) - cyclic guanosine monophosphate (cGMP) pathway (PDE5I or riociguat) [28]. In the AMBITION trial, the combination of ambrisentan and tadalafil resulted in the reduction of clinical failure (number of hospitalizations) and improved exercise capacity when compared to either agent alone. Interestingly, the rates of hypotension were similar in all groups [29]. The combination of riociguat and sildenafil (a PDE5I) is associated with hemodynamically significant hypotension leading to the discontinuation of therapy. In the GRIPHON trial, in patients with PAH (almost 30% in both groups had PAH associated with connective tissue disease), the addition of selexipag (an oral selective non-prostanoid prostacyclin receptor agonist) was associated with a decreased risk of the primary composite endpoint of death or a complication related to PAH when compared to placebo. However, no difference in mortality was observed between the two groups [30]. An intravenous administration of epoprostenol was found to improve exercise capacity and hemodynamics in patients with moderate-to-severe SSc-PAH when compared to placebo.

Lung transplant

Indications

Regardless of the etiology of advanced lung disease, there are certain criteria that have been developed for the selection of appropriate recipients for LT. The most recent guidelines were published by the International Society for Heart and Lung Transplantation (ISHLT) in 2014. All of the following criteria should be met in order to be considered for LT: (1) high (>50%) risk of death from lung disease within two years if LT is not performed; (2) high (>80%) likelihood of surviving at least 90 days after LT; and (3) high (>80%) likelihood of a five-year post-transplant survival from a general medical perspective provided that there is an adequate graft function. The authors of the proposed guidelines have mentioned that LT in patients with SSc remains controversial, mostly because of the risk of aspiration in patients with esophageal dysmotility [31]. As stated above, ILD and PAH are the most common pulmonary manifestations of SSc. In general, patients with ILD should be listed for LT if any of the following indications are present: (1) decline in FVC of ≥10% during a six-month follow-up; (2) decline in DLCO of ≥15% during a six-month follow-up; (3) desaturation to <88% or distance of <250 m on a six-minute walk test (6MWT) over a six-month follow-up period; and (4) RHC or echocardiogram findings consistent with PH. Similarly, there are certain criteria that are used to list patients with PAH for LT: (1) NYHA functional class III and IV despite a trial of at least three months of combination therapy including prostanoids; (2) cardiac index of <2 liters/min/m^2^; (3) mean right atrial pressure of >15 mmHg; (4) 6MWT of <350 m; and (5) significant hemoptysis, pericardial effusion, or signs of progressive right heart failure. Unfortunately, to this date, LT in patients with SSc remains controversial since no criteria have been defined in light of the various challenges.

Contraindications

Although there are no universally accepted contraindications to LT in patients with SSc, the following comorbidities are reportedly considered to be contraindications: (1) skin breakdown (increased risk of infection); (2) renal failure with a creatinine clearance of <50 ml/min; (3) severe esophageal dysmotility and gastroparesis (risk of aspiration); and (4) significant cardiac involvement (conduction abnormalities). The lack of specific contraindications makes the process of selecting SSc patients appropriate for LT a challenging task. Hence, intercenter variability in defining absolute and relative contraindications is widely recognized. 

Perioperative Evaluation

After thorough patient selection based on the combination of indications and contraindications, all the eligible patients should undergo a comprehensive perioperative evaluation. It is worth mentioning that there are no universally accepted protocols to assess these patients. A multidisciplinary team approach is used in many institutions. Extrapulmonary manifestations (GI, renal, skin, cardiac) of SSc remain a significant challenge in the perioperative care of potential LT candidates.

Gastrointestinal: it is estimated that most patients with SSc develop some degree of esophageal involvement. Its symptoms are mostly attributed to either an alteration of esophageal peristalsis or lower esophageal sphincter incompetence. Gastroesophageal reflux disease (GERD) increases the risk of aspiration and, therefore, has been linked to the development of BOS. The latter is manifested as a progressive drop in the forced expiratory volume in one second (FEV1). Notably, aspiration-induced BOS has been associated with post-transplant chemotherapeutic agents (via delayed gastric emptying) as well as iatrogenic intraoperative vagal nerve injury. Other important GI manifestations that may complicate the postoperative period and affect survival include intestinal involvement (small intestinal bacterial overgrowth syndrome, chronic GI bleeding, intestinal fibrosis). Thorough GI evaluation in SSc patients is highly recommended. Although no consensus guidelines are available at this point, many institutions perform several tests, including upper GI barium swallow, dual pH probe study, esophageal manometry, esophagogastroduodenoscopy (EGD), and gastric emptying study. High-dose proton pump inhibitor therapy is employed in the postoperative period regardless of the degree of esophageal dysfunction. Strict enteral feeding (occasionally post-pyloric gastric) is recommended in all patients after LT for several months until oral feeding is restarted after the improvement of swallowing function. Paradoxically, surgical correction (primarily Nissen fundoplication), despite improving reflux disease, may increase the risk of aspiration by means of altering antegrade emptying of the esophagus.

Skin: most of the SSc patients evaluated for LT have cutaneous manifestations of the disease ranging from skin induration to rapidly progressive diffuse skin thickening. The latter as well as digital ulceration (with concern for digital gangrene) are considered contraindications for LT. In addition, severe skin involvement of the chest can make the healing process in the postoperative period challenging and requires a comprehensive perioperative evaluation.

Renal: renal involvement in SSc has been widely recognized due to its significant morbidity and mortality. As mentioned above, SRC was once a leading cause of death in patients with SSc. With the introduction of ACEIs, the prognosis in patients with SRC has significantly improved. Patients undergoing LT are required to have preserved renal function (creatinine clearance of >50 ml/min) for at least three months prior to the surgery. In addition, some experts recommend delaying enlisting for LT for at least five years after the diagnosis of SRC. Steroids and other immunosuppressive medications have been associated with an increased risk of SRC. However, it remains unclear whether SSc patients undergoing LT should receive ACEIs for SRC prophylaxis in the postoperative period.

Cardiac: PAH affecting cardiovascular performance is a well-known complication of SSc. Apart from that, in a majority of autopsy specimens of patients with SSc, patchy myocardial fibrosis is also present. Pericarditis, myocarditis, and arrhythmias (both supraventricular and ventricular) in patients with SSc have been described in the literature. In the preoperative period, patients should undergo a comprehensive cardiac evaluation including an electrocardiogram (EKG), a 24-hour Holter monitoring, an echocardiogram, right and left cardiac catheterizations. Recently, cardiac MRI has been frequently obtained in the pre- and post-transplant cardiac assessment to better evaluate structural cardiac disease (fibrosis, inflammation) in patients with SSc. Interestingly, in SSc patients with right heart failure who undergo LT for PAH, right ventricular function tends to improve in the postoperative period [32]. In patients without PAH, preserved biventricular systolic function is required for LT consideration. To this date, there have been very few cases of combined heart and lung transplants. Therefore, taking care of patients with SSc with compromised cardiovascular performance requires the application of clinical judgment to determine whether the benefits of undergoing LT outweigh the risks of perioperative cardiovascular mortality.

Single vs. Bilateral LT

There has never been a randomized controlled study comparing unilateral vs. bilateral LT. As a general rule, in patients with the risk of suppurative lung disease (cystic fibrosis or bronchiectasis), bilateral LT is the only acceptable method given the risk of infectious complications in the donor's lung in the case of a single LT. In SSc patients, bilateral LT is usually preferred in patients younger than 60 years of age and those with severe PAH. Although there are no strict guidelines regarding which method should be used, many centers do prefer to perform bilateral LT. Unfortunately, most of the survival data may not accurately reflect survival differences in patients undergoing single vs. bilateral LT given the presence of multiple biases (most importantly, selection bias).

Survival after LT in patients with SSc

Methods

We conducted a comprehensive literature search on PubMed/Medline to identify all the articles that have reported data on survival after LT in patients with SSc. We used the following search terms: “scleroderma”, “systemic sclerosis”, and “lung transplant”. The Boolean operators “OR” (“scleroderma”, “systemic sclerosis”) and “AND” (“lung transplant”) were selected to specify the search outcome. All articles since inception until December 2020 were included in the literature search. We included only those articles that had abstracts in English. Out of 27 identified articles, only 11 were included for further review. The remaining 16 articles were excluded due to the following factors: (1) the lack of data on patient survival after LT (including articles where LT was not evaluated); (2) evaluation of non-LT-related complications of SSc; (3) no patients with SSc were included in the study; (4) focus on the survival outcomes related to the use of additional therapeutic interventions; and (5) inclusion of only those patients who had post-transplant serial imaging tests (could significantly confound the true survival in those patients).

**Figure 1:**
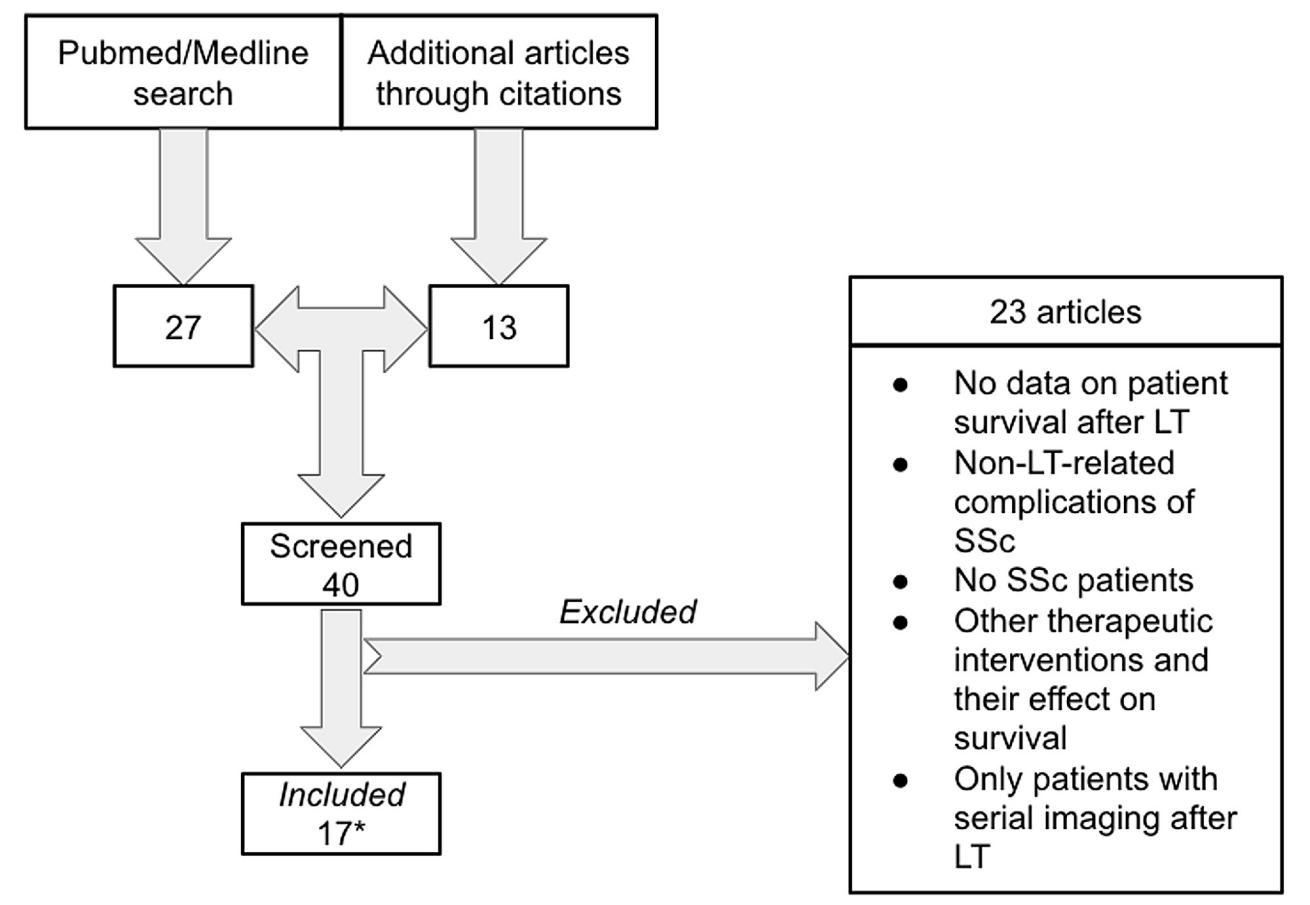
Flow diagram of the literature search *[33-49] LT: lung transplant; SSc: systemic sclerosis

In addition to the 11 articles that were included for further review, we were able to identify another 13 articles via references. With further manual verification of data relevance, only 17 articles were included for data collection and analysis (Figure 1).

Results

All of the included articles were retrospective ones with the majority of them having been conducted at a single institution (single center: 13 [33-45], two centers: one [46], multicenter: three [47-49]) (Table 1). The US-based publications represented the majority of the collected data (11 out of 17). The remaining six were from Israel (one), China (one), Canada (two), and Europe (two). All of the patients were enrolled between 1983 and 2017. A total number of 6,057 patients underwent LT in the reported 17 studies [712 patients with SSc and the remaining 5,355 patients with other indications for LT (see "Control group" in Table 1)]. The average number of patients enrolled in the studies was 330.4 (ranging between seven and 3,763). In five studies, which included 166 patients, no control groups (patients without SSc) were included.

The reported mean age of the subjects varied between 46 and 63.1 years. The type of transplant was reported in a majority of the articles (16 out of 17; 6,036 patients). Interestingly, in patients with SSc, bilateral LT was performed almost four times more often than single LT (537 vs. 139 patients, respectively). Notably, in control groups the difference in the prevalence of bilateral LT when compared to single LT was minimal (2,822 vs. 2,493 patients). A small percentage of patients in both groups underwent a combined heart and lung transplant (HLT): 17 (~2.4%) patients in the SSc group and 18 (~0.3%) in the control group. In seven studies, the subgroups of SSc were available (226 patients) - lcSSc: 124, dcSSc: 100, and sine scleroderma: two. The indications for LT in patients with SSc were reported only in 11 studies (488 patients). The majority of patients had SSc-ILD subtype (216), followed by SSc-PAH (177) and SSc-ILD-PAH overlap (95). Although the presence and severity of GERD have been recognized as an important prognostic factor in LT recipients, only five studies provided data on the prevalence of GERD in their study subjects. Patients with severe GERD were excluded in two studies. Interestingly, Crespo et al. included patients with moderate-to-severe esophageal dysmotility that was four times more prevalent in patients with SSc compared to controls (83.3% vs. 20.6%) [38]. Similarly, in another study (Miele et al.), patients with SSc were more likely to have a severe esophageal dysfunction than a matched cohort with diffuse fibrotic lung disease (54% vs. 8%) [39].

**Table 1:** The survival in patients with systemic sclerosis undergoing lung transplantation (literature review) *P-value of <0.05 when compared to SSc; **not statistically significant difference SSc: systemic sclerosis; IPF: idiopathic pulmonary fibrosis; IPAH: idiopathic pulmonary arterial hypertension; SLT: single-lung transplant; BLT: bilateral lung transplant; HLT: heart-lung transplant; SSc-ILD: systemic sclerosis, interstitial lung disease subtype; SSc-PAH: systemic sclerosis, pulmonary arterial hypertension subtype; SSc-ILD-PAH: systemic sclerosis, overlap between interstitial lung disease and pulmonary arterial hypertension; dcSSc: diffuse cutaneous systemic sclerosis; lcSSc: limited cutaneous systemic sclerosis; CTD: connective tissue disease; DFLD: diffuse fibrotic lung disease; COPD: chronic obstructive pulmonary disease; RA-ILD: rheumatoid arthritis-associated interstitial lung disease; PM/DM-ILD: polymyositis/dermatomyositis-associated interstitial lung disease; pSS-ILD: primary Sjögren's syndrome-associated interstitial lung disease; NR: not reported; HR: hazard ratio; CI: confidence interval

Author (country, year)	Enrollment period (years)	Type of study	Number of patients	Mean age in years	Type of transplant (SLT vs. BLT)	Subgroups of SSc patients (dcSSc, lcSSc)	Indication for the lung transplant in SSc patients (ILD, PAH)	Control group	Presence of GERD	Survival: 30 days	Survival: 6 months	Survival: 1 year	Survival: 2 years	Survival: 3 years	Survival: 4 years	Survival: 5 years	Reference	Comments
Massad et al. (the USA, 2005) [47]	1987-2004	Retrospective, multicenter	47	46	SLT: 27; BLT: 20	NR	NR	N/A	NR	85%	NR	67.60%	49%	NR	NR	NR	87	
Schachna et al. (the USA, 2006) [46]	1989-2002	Retrospective, two centers	137 (SSc: 29; IPF: 70; IPAH: 38)	SSc: 46.4; IPF: 55.7*; IPAH: 41.5*	SSc – SLT: 18, BLT: 9, HLT: 2; IPF – SLT: 66, BLT: 4; IPAH – SLT: 29, BLT: 5, HLT: 4	lcSSc: 17; dcSSc: 12	SSc-ILD: 15; SSc-PAH: 11; SSc-ILD-PAH: 3	108 (IPF: 70; IPAH: 38)	NR (patients with severe GERD were excluded)	SSc: 76%; IPF: NR; IPAH: NR	SSc: 69%; IPF: 89%**; IPAH: 79%** (not statistically different)	NR	SSc: 62%; IPF: 67%**; IPAH: 63%**	NR	NR	NR	86	
Shitrit et al. (Israel, 2009) [35]	1997-2006	Retrospective, single-center	7	52	All patients underwent SLT	NR	SSc-ILD: 1; SSc-ILD-PAH: 6	N/A	NR	NR	NR	88%^	NR				75	^Similar to non-SSc lung recipients at the same center, 84%
Saggar et al. (the USA, 2010) [34]	2003-2007	Retrospective, single-center	52 (SSc: 14; IPF: 38)	SSc: 58.8; IPF: 53.2*	All patients underwent BLT	NR	SSc-PAH: 2; SSc-ILD: 6; SSc-ILD-PAH: 6	IPF: 38	NR (patients with severe GERD were excluded)	NR	NR	SSc: 93.4%; IPF: 86.9%**	SSc: 80%; IPF: 71/1%**				66	
Sottile et al. (the USA, 2013) [36]	1998-2012	Retrospective, single-center	69 (SSc: 23; non-CTD-ILD: 46)	SSc: 49.3; non-CTD-ILD: 51.5	All patients underwent BLT	lcSSc: 17; dcSSc: 4; SSSS: 2	ILD: 23	Non-CTD-ILD: 46	SSc: 52.2%; non-CTD-ILD: 41.3%**			SSc: 83%; non-CTD-ILD: 91%**		SSc: 83%; non-CTD-ILD: 77%**		SSc: 76%; non-CTD-ILD: 64%**	76	
Launay et al. (France, 2014) [37]	1993-2012	Retrospective, single-center	13	48	SLT: 1; BLT: 7; HLT: 5	lcSSc: 11; dcSSc: 2	SSc-ILD: 3; SSc-PAH: 8; SSc-ILD-PAH overlap: 2	N/A	All patients. 5 patients had severe GERD			62%		59%			77	
Bernstein et al. (the USA, 2015) [48]	2005-2012	Retrospective, multicenter	3,763 (SSc: 229; PAH: 201; ILD: 3,333)	SSc: 53^§^;PAH: 46^§^; ILD: 62^§^	SSc – SLT: 54, BLT: 175; PAH – SLT: 8, BLT: 193; ILD – SLT: 1,673, BLT: 1,660	NR	SSc-PAH: 123; SSc-ILD: 105; could not be determined: 1	3,534 (PAH: 201; ILD: 3,333)	NR	^¥^	NR	SSc: 81.2%^†^; PAH: 83.6%^†^;ILD: 84.2%^†^	NR	NR			88	^§^Median age; ^¥^adults with SSc did not have a multivariable-adjusted increased risk of death compared to ILD [HR: 0.65, 95% CI: (0.27-1.58)], but 78% relative decrease in the risk of death when compared to PAH [HR: 0.22, 95% CI: (0.08-0.64)]; ^†^adults with SSc had a 48% multivariate-adjusted relative increase in the mortality rate compared to ILD [HR: 1.38, 95% CI: (1.01-2.17)], but no difference when compared to PAH [HR 0.85, 95% CI: (0.50-1.44)]
Crespo et al. (the USA, 2015) [38]	2005-2013	Retrospective, single-center	383 (SSc: 72; ILD: 311)	SSc: 52.3; ILD: 63.1*	SSc – BLT: 65, SLT: 7; ILD – BLT: 172*, SLT: 139*	lcSSc: 38; dcSSc: 34	SSc-ILD: 34; SSc-ILD-PAH: 38	ILD: 311	Moderate-to-severe esophageal dysmotility was more common in patients with SSc compared to ILD (83.3% vs. 20.6%)*	SSc: 100%; ILD: 96.3%**		SSc: 80.6%; ILD: 78.8%**				Conditional on 1-year survival – SSc: 66%; ILD: 58%**	78	
Miele et al. (the USA, 2016) [39]	2000-2012	Retrospective, single-center	562 (SSc: 35; non-SSc: 527)	SSc: 50.7; non-SSc: 58.5, including – DFLD: 61.8, matched group: 53.9^£^	SSc – BLT: 32, SLT: 3; non-SSc – BLT: 266, SLT: 261; DFLD – BLT: 105, SLT: 159; matched group – BLT: 97, SLT: 12	NR	NR	527 (non-SSc) including DFLD (264) and matched group (109)	In comparison to DFLD subgroup of the matched group, patients with SSc had more severe esophageal dysfunction (54% vs. 8%)*			SSc: 94%; non-SSc: 88%**; DFLD: 84%**; matched group: 92%**		SSc: 77%; non-SSc: 68%**; DFLD: 64%**; matched group: 71%**		SSc: 70%; non-SSc: 54%**; DFLD: 49%**; matched group: 60%**	79	^£^Non-SSc patients matched to SSc patients (4:1) through Greedy distance matching including age, lung allocation score, transplant type, and pulmonary hypertension
Pradère et al. (Europe, 2018) [49]	1993-2016	Retrospective, multicenter	90	49	SLT: 15; BLT: 66; HLT: 9	Data were available only for 62 patients – lcSSc: 27; dcSSc: 35	SSc-ILD: 30; SSc-PAH: 20; SSc-ILD-PAH: 40	N/A	16% of patients had severe GERD			81%		68%		61%	89	Factors associated with worse survival included female gender and presence of PAH
Chan et al. (the USA, 2018) [45]	2006-2014	Retrospective, single-center	181 (SSc: 26; non-SSc group D restrictive disease: 155)	SSc: 54; non-SSc group D restrictive disease: 60*	All patients underwent BLT	NR	NR	Non-SSc group D restrictive disease: 155	NR	SSc: 88.5%; non-SS group D restrictive disease: 95.5%**	SSc: 80.8%; non-SS group D restrictive disease: 87.7%**	SSc: 73.1%; non-SS group D restrictive disease: 80%**		SSc: 69.2%; non-SS group D restrictive disease: 69.7%**		SSc: 65.4%; non-SS group D restrictive disease: 66.5%**	85	
Pakhale et al. (Canada, 2002). Abstract only [40]	1983-2001	Retrospective, single-center	9	47	SLT: 2; BLT: 7	NR	NR	N/A	NR	88.90%							80	
Kubo et al. (the USA, 2001). Abstract only [41]	1990-2000	Retrospective, single-center	138 (SSc: 12; COPD: 105; IPF: 21)	SSc: 47.5	SSc – SLT: 10, BLT: 2; COPD and IPF – all underwent SLT	NR	NR	COPD: 105; IPF: 21	NR	SSc (total): 75%		SSc (total): 75%; SSc (those who underwent SLT): 80%; COPD: 76%; IPF: 71%		SSc (total): 67%		SSc (total): 50%; SSc (those who underwent SLT): 52%; COPD: 47%; IPF: 49%	81	
Rosas et al. (the USA, 2000). Abstract only [33]	1994-2000	Retrospective, single-center	31 (SSc: 9; IPF: 12; PAH: 10)	NR	NR	lcSSc: 6; dcSSc: 3	SSc-ILD: 4; SSc-PAH: 4; Unsure as to how many patients had an overlap between ILD and PAH	IPF: 12; PPH: 10	NR. Of note, patients with aspiration were excluded						SSc: 76.2%; non-SSc: 69.2%**		60	
Yazdani et al. (Canada, 2014) [42]	2011-2014	Retrospective, single-center	80 (SSc: 17; RA-ILD: 10; IPF: 53)	SSc: 45.4; RA-ILD: 59.4*; IPF: 61*	SSc – SLT: 3, BLT: 13, HLT: 1; RA-ILD – SLT: 3, BLT: 6, HLT: 1; IPF – SLT: 13, BLT: 40	NR	SSc-ILD: 17	RA-ILD: 10; IPF: 53	NR			SSc: 82%; RA-ILD: 67%**; IPF: 69%**					82	
Gadre et al. (the USA, 2017) [43]	1992-2013	Retrospective, single-center	51 (SSc-PAH: 9; non-SSc-PAH: 42)	SSc-PAH: 52.6; non-SSc-PAH: 40.3*	SSc-PAH – SLT: 2, BLT: 7; non-SSc-PAH – SLT: 3, BLT: 26, HLT: 13	lcSSc: 8; dcSSc: 1	SSc-PAH: 9	Non-SSc-PAH: 42 [idiopathic: 31; congenital heart disease-associated: 9; connective tissue disease-associated: 2 (SLE and RA)]	NR			SSc-PAH: 100%; non-SS-PAH: 86.8%**	SSc-PAH: 71.4%; non-SS-PAH: 76.3%**			SSc-PAH: 14.3%; non-SSc-PAH: 47.4%**	83	Prior to transplantation, patients with non-SSc-PAH had higher estimated RV systolic pressure, severe RV systolic dysfunction, and overall worse pulmonary hemodynamics by right heart catheterization when compared to SSc-PAH (all with a statistically significant difference)
Zhang et al. (China, 2017) [44]	2015-2017	Retrospective, single-center	11 (SSc-ILD: 1; PM/DM-ILD: 2; RA-ILD: 4; pSS-ILD: 4)	52.7	SSc – BLT: 1; control group – BLT: 9, SLT: 1	NR	SSc-ILD: 1	Non-SSc-ILD – PM/DM: 2, RA: 4, pSS: 4. Reportedly, 6 patients had ILD-PAH overlap	NR	NR	NR	NR	NR	NR	NR	NR	84	Reportedly, 3 patients died in the early postoperative period. The remaining 8 patients were followed up for 4-24 months with "good" survival. "The survival rate was comparable to that of other disease sources"

Survival data

Across all studies, the survival was reported at different time intervals: 30 days, six months, and one to five years. In a majority of the studies, one-year survival was mentioned by the authors. There was no statistical difference in the post-LT survival of patients with SSc when compared to control groups. Surprisingly, even when the prevalence of moderate-to-severe esophageal dysmotility was more common in patients with SSc when compared to the control group (Crespo et al.), one-year survival was better in SSc patients, although it was not statistically significant (80.6% vs. 78.8%, p=0.743) [38]. Overall, one-year survival ranged between 62% and 100% for SSc across all studies. For control groups, the range was between 67% and 92%. Long-term survival (at five years) was reported in seven studies. Again, there was no statistically significant difference between SSc patients and control groups. Nevertheless, in most of the reported studies, the survival in SSc patients was better, although not statistically significant. However, Gadre et al. published quite interesting survival data on their subjects. In their study, patients with SSc (all had PAH subvariant) had much worse survival at five years compared to non-SS PAH patients (14.3% vs. 47.4%, not statistically significant). The authors speculated that the lack of power (only nine patients in the SSc group vs. 42 patients in the non-SSc-PAH group) may have resulted in the statistically non-significant difference. Interestingly, prior to LT, the control group had higher estimated right ventricular systolic pressure (RVSP), severe RV systolic dysfunction, and overall worse pulmonary hemodynamics by RHC, when compared to the SSc group (all with a statistically significant difference). Despite that, one-year survival was comparable between the two groups (100% for the SSc patients, 86.8% for the control group). After adjusting for multiple variables, the male gender and severe tricuspid regurgitation before lung transplantation were found to be independent predictors of poor post-LT survival [43].

## Conclusions

Despite the lack of universally accepted protocols for LT in patients with SSc, more data have emerged supporting the safety and efficacy of transplant approach in patients with pulmonary manifestations of SSc. Patients with SSc who undergo LT (even with moderate-to-severe esophageal dysmotility) have shown survival outcomes similar to those of patients without SSc. Medical professionals should be encouraged to promptly refer patients to specialized LT centers when features of progressive pulmonary disease are suspected. The establishment of standardized protocols for referral and step-by-step assessment of SSc patients requiring lung transplant are warranted. Furthermore, prospective multicenter studies with the adoption of standardized perioperative evaluation of SSc patients should be conducted in the future.
